# A Novel One-Step Hydrothermal Preparation of Ru/Sn_*x*_Ti_1−*x*_O_2_ Diesel Oxidation Catalysts and its Low-Temperature Performance

**DOI:** 10.1186/s11671-020-03339-4

**Published:** 2020-05-14

**Authors:** Li Fan, Qi Sun, Wei Zheng, Qinyuan Tang, Ting Zhang, Mengkui Tian

**Affiliations:** School of Chemistry and Chemical Engineering, Guizhou University, Guiyang, 550025 China

**Keywords:** Solid solution, Rutile, One-step hydrothermal method, Low-temperature activity, Stability

## Abstract

The rutile Sn_*x*_Ti_1−*x*_O_2_ (*x* = 0, 0.33, 0.5, 0.67, 1) solid solution was synthesized by a one-step hydrothermal method, in which tetrabutyl titanate and Tin (IV) chloride pentahydrate were used as raw materials. A series of Ru/Sn_*x*_Ti_1−*x*_O_2_ were then prepared by the impregnation process in RuCl_3_ to investigate the performance and stability of CO and C_3_H_8_ oxidation. These catalysts were characterized through XRD, N_2_ adsorption-desorption, FT-IR, TEM, XPS, H_2_-TPR, and O_2_-TPD techniques. The effect of Sn/Ti molar ratio and hydrothermal condition on the low-temperature catalytic oxidized performance and stability of Ru/Sn_*x*_Ti_1−*x*_O_2_ were investigated. The results indicated that Ru/Sn_0.67_Ti_0.33_O_2_ catalyst showed an excellent activity and stability at low temperatures. The CO conversion reached 50% at 180 °C and 90% at 240 °C. Besides, the C_3_H_8_ conversion reached 50% at 320 °C, the complete conversion of C_3_H_8_ realized at 500 °C, and no deactivation occurs after 12 h of catalytic reaction. The excellent low-temperature activity and stability of the Ru/Sn_0.67_Ti_0.33_O_2_ were attributed to the following factors. Firstly, XRD results showed that Sn^4+^ was successfully introduced into the lattice of TiO_2_ to replace Ti^4+^ forming a homogeneous solid solution (containing –Sn^4+^–O–Ti^4+^– species), which was consistent with TEM and N_2_ adsorption-desorption results. The introduction of Sn could suppress the growth of anatase crystal and promote the formation of rutile phase, and this phase transition was helpful to improve the low-temperature activity of the catalysts. Secondly, TEM images showed that ultrafine Ru nanoparticles (~ 5 nm) were dispersed on Sn_0.67_Ti_0.33_O_2_ support, suggesting that the formation of Sn_*x*_Ti_1−*x*_O_2_ solid solution was beneficial to the dispersion of Ru particles.

## Background

Diesel engines are widely applied in the field of transportation, mining, and engineering machinery due to these advantages of low fuel consumption, high thermal efficiency, and good stability [1]. However, carbon monoxide (CO), unburned hydrocarbons (HCs), various oxides of nitrogen (NO_*x*_), and the particulate matter (PM) in diesel vehicle exhaust have caused a serious threat to ecological environment and human health [2, 3]. Furthermore, stringent environmental laws and regulations have driven recent advances in diesel emission control technologies. An integrated exhaust after-treatment system consisting of diesel oxidation catalyst (DOC), selective catalytic reduction (SCR), and catalyzed diesel particulate filter (DPF) has been widely used to purify diesel exhausts. The functions of the DOC in the after-treatment system are converting CO, HCs, and NO into CO_2_, H_2_O, and NO_2_, the NO_2_ being used as raw material for subsequent de-NO_*x*_ reaction to promote SCR reaction. In addition, it could also oxidize the soluble organic fraction (SOF) to decrease PM emissions. HCs excessive emission will be caused owing to the incomplete combustion of HCs during the cold start of diesel vehicles. Therefore, the catalysts need to ignite rapidly at low temperatures [4]. Presently, noble metal catalysts (such as Pt, Pd, and Rh) supported on carbon materials or oxides (such as TiO_2_, Al_2_O_3_, CeO_2_, and ZrO_2_) are commercialized diesel oxidation catalysts with good performance for CO, NO, and HCs catalytic oxidation. However, there are drawbacks to commercialized catalysts, such as poor thermal stability, strong self-inhibition by CO, and high cost [5].

Ru and RuO_*x*_ catalysts are widely applied in oxidizing CO [6], methane [7], and chlorobenzene [8]. Importantly, Ru catalysts have excellent low-temperature activity and poison resistance [8–11]. But Ru and RuO_*x*_ are easily sintered, resulting in active sites’ exposure decreases [12]. Therefore, Ru catalysts should be supported on a carrier to prevent their sintering and improve catalytic activity.

TiO_2_ has been widely used to purify diesel exhausts. RuO_*x*_ and rutile phase TiO_2_ have a similar lattice constant, and the rutile TiO_2_ in Ru/TiO_2_ catalysts plays an important role in stabilizing RuO_*x*_ particles during calcination process in comparison with anatase-supported RuO_*x*_ catalysts. Therefore, RuO_*x*_ can be highly dispersed on the surface of TiO_2_. Furthermore, there is a synergistic effect between RuO_*x*_ and TiO_2_, which is beneficial to improve the redox ability of Ru/TiO_2_ [13–18]. In order to further improve the thermal stability, dispersion of active components, and transformation of anatase to rutile phase, many studies have introduced Sn^4+^ into TiO_2_ to form Sn_*x*_Ti_1−*x*_O_2_ solid solution. Huang et al. [16] found that the introduction of Sn^4+^ into TiO_2_ lattice could improve the stability of the CuO/Ti_*x*_Sn_1−*x*_O_2_ catalysts and dispersion of CuO. Bai et al .[17] indicated Sn^4+^ significantly improved the thermal stability of TiO_2_. Mehraz et al. [18] found doping Sn^4+^ promoted the phase transition of TiO_2_ from anatase to rutile.

Previous researches have focused on the preparation of diesel oxidation catalysts by co-precipitation method, sol-gel method, and solid-phase reaction [5, 6, 15, 19, 20]. Yang et al. [19] prepared the Pt/TiO_2_ catalysts via the co-precipitation method and found that the conversion of CO and C_3_H_6_ only reaches 50% at 232 °C. Li et al. [15] synthesized TiO_2_–SnO_2_ nano-composite by the sol-gel method and suggested that the conversion of TiO_2_–SnO_2_ to CO was 90% at 260 °C. Sharif et al. [6] prepared Ru/[Ca_24_Al_28_O_64_]^4+^(O^2−^)_2_ through solid-state reaction and showed that the conversion of Ru/[Ca_24_Al_28_O_64_]^4+^(O^2−^)_2_ to CO was only 82% at 240 °C due to lower dispersion of Ru. Therefore, there are critical challenges that remain in the low-temperature activity of diesel oxidation catalysts and a lot of efforts are still needed to remove CO and HCs caused in the diesel cold start. Furthermore, the current research [8, 16, 19, 21, 22] is mainly focused on the preparation of DOC catalysts by co-precipitation and sol-gel methods, which has a small grain size, but the samples have poor crystallinity and multiple crystal phases; furthermore, the subsequent heat treatment process of mixture by co-precipitation method is required. Hydrothermal treatment is adopted in the preparation process to avoid the traditionally followed calcination processes and the formation of hard aggregation of the catalysts, which could improve low-temperature catalytic activity [23]. However, there is a lack of systematic and comprehensive studies on the one-step hydrothermal method [24, 25].

Therefore, we reported that RuO_*x*_ particles supported on the Sn^4+^-modified TiO_2_ by the one-step hydrothermal method were excellent CO and HC oxidation catalysts with promising low-temperature activity and stability. A series of Sn_*x*_Ti_1−*x*_O_2_ (*x* = 0, 0.33, 0.5, 0.67, 1) solid solution were prepared by the one-step hydrothermal method. Ru/Sn_*x*_Ti_1−*x*_O_2_ were then prepared by impregnation of the Sn_*x*_Ti_1−*x*_O_2_ with RuCl_3_ to oxidize CO and C_3_H_8_. The effect of hydrothermal temperatures, hydrothermal time, calcination temperatures, and the molar ratios of Sn/Ti of Ru/Sn_*x*_Ti_1−*x*_O_2_ catalysts were investigated in order to improve low-temperature activity and stability.

## Method

### Materials

Tin (IV) chloride pentahydrate (SnCl_4_·5H_2_O) was purchased from Guangdong Kehua Stock Corporation, tetrabutyl titanate (C_16_H_36_O_4_Ti) was purchased from Tianjin Kemiou Chemical Reagent Factory, and Ruthenium (III) chloride anhydrous, RuCl_3_, (37% Ru w/w) was purchased from Aladdin.

### Preparation of Catalysts

Sn_*x*_Ti_1−*x*_O_2_ solid solution was prepared by the one-step hydrothermal method. The certain amounts of SnCl_4_·5H_2_O and C_16_H_36_O_4_Ti were dissolved in 200 mL of deionized water and 10 mL of anhydrous ethanol, respectively; then, C_16_H_36_O_4_Ti ethanol solution and SnCl_4_·5H_2_O aqueous solution were mixed while stirring at room temperature for 0.5 h. The homogeneous mixture was put in a 250-mL autoclave at 180 °C for 24 h. After that, the mixed solution was centrifuged washing with deionized water and ethanol several times until no residues of Cl^−^, and then was dried at 80 °C overnight in the oven. Subsequently, light yellow solid products were obtained, named Sn_*x*_Ti_1−*x*_O_2_. SnO_2_ and TiO_2_ were obtained by similar preparation methods, respectively.

Ru/Sn_*x*_Ti_1−*x*_O_2_ catalysts were prepared by impregnation of Sn_*x*_Ti_1−*x*_O_2_ with an aqueous solution including 1.0 wt.% of RuCl_3_. These samples were ultrasonic stirred for 2 h and dried at 80 °C for 12 h, and it was then calcined at 400 °C for 3 h (heating rate is 3 °C/min). The obtained powder was named Ru/Sn_*x*_Ti_1−*x*_O_2_.

### Catalytical Performance

The activities of the catalysts were evaluated on a fixed bed quartz reactor with an electric heater. The simulative reactant gases contained a mixture of 3000 ppm CO, 600 ppm C_3_H_8_, 600 ppm NO, 50 ppm SO_2_, 7% O_2_, and N_2_ balance at a gas space velocity of 60,000 mL g^−1^ h^−1^. The gas flow rate was regulated by mass flow controllers. The temperature of fixed bed was tested by a 0.5-mm K-thermocouple which was placed in the middle of the center channels. The outlet CO and C_3_H_8_ were measured by a KM9106 flue gas analyzer (Kane International Limited, Britain). The conversion (*X*) of CO and C_3_H_8_ was calculated using the following equation:

$$ X=\frac{c_{\mathrm{in}}-{c}_{\mathrm{out}}}{c_{\mathrm{in}}}\times 100\% $$

where *c*_in_ is the initial concentration of CO or C_3_H_8_ and *c*_out_ is the instantaneous of CO or C_3_H_8_ at the reaction temperature; *T*_50_ is denoted as the low-temperature catalytic activity index.

### Catalyst Characterization

X-ray diffraction (XRD) patterns of the samples were performed by power X-ray diffraction on a BRUKER D8 ADVANCE diffractometer equipped with a high-temperature chamber using Cu Kα radiation (0.15418 nm). The X-ray tube was operated at a source power of 40 kV × 40 mA.

The Brunauer-Emmett-Teller (BET) surface areas were tested by nitrogen adsorption at 77 K on a Micromeritics ASAP2020 adsorption apparatus; the specific surface area and pore distribution were calculated by the BET and BJH methods, respectively. These samples were degassed under vacuum at 300 °C for 4 h before each analysis.

Fourier transform infrared (FT-IR) spectroscopy was examined using a Nicolet is5 spectrometer at a spectral resolution of 4.0 cm^−1^. The powders were pressed into a self-supporting wafer (about 15 mg, 12 mm diameter). The wafer was pretreated with N_2_ at 300 °C for 1 h. After cooling to ambient temperature, the spectrum of samples was recorded.

Transmission electron microscopy (TEM) images of these samples were obtained by a Tecnai G2 F20 instrument at an acceleration voltage of 200 kV. The samples were ground, dispersed in ethanol, and deposited on carbon-coated copper grids prior to observation.

X-ray photoelectron spectroscopy (XPS) analysis was performed on a ESCALAB250Xi spectrometer, using monochromatic Al Kα radiation (1486.6 eV) at an accelerating power of 15 kW. The obtained sample spectra were corrected using C1s (284.6 eV) as the internal reference standard.

H_2_-temperature-programmed reduction (H_2_-TPR) experiments were performed in a quartz reactor connected to a thermal conductivity detector (TCD) with H_2_ (6.9% vol. %)-Ar mixture (30 mL/min) as reductant. Prior to the reaction, the sample (50 mg) was pretreated in N_2_ at 300 °C for 1 h and then cooled to room temperature. TPR started from room temperature to target temperature at a rate of 10 °C/min.

Temperature-programmed oxygen desorption (O_2_-TPD) experiments were carried out using the same device as H_2_-TPR. The spent catalyst (50 mg) was pretreated at 300 °C for 1 h under flowing Ar at 30 mL/min. Then, oxygen adsorption was conducted under an O_2_–Ar mixture (20% O_2_ vol. %) at 500 °C for 0.5 h. After cooling to room temperature, the system was purged in Ar (30 mL/min) for 1 h. After the treatment, the temperature was raised to target temperature (10 °C/min).

In situ infrared spectroscopy (IR) of CO adsorption was collected on a Nicolet 5700 FT-IR spectrometer at a spectral resolution of 4.0 cm^−1^. CO adsorption was performed by exposing a self-supporting wafer of catalyst (about 15 mg) and mounted in a commercial controlled environment chamber (HTC-3). The samples were exposed to a controlled stream of CO–Ar (10% of CO by volume) at a rate of 5.0 mL/min for 40 min. The spectra were recorded at various target temperatures at a rate of 10 °C/min from room temperature to 300 °C.

## Results and Discussion

### Catalytic Activity and Stability

**Fig. 1:**
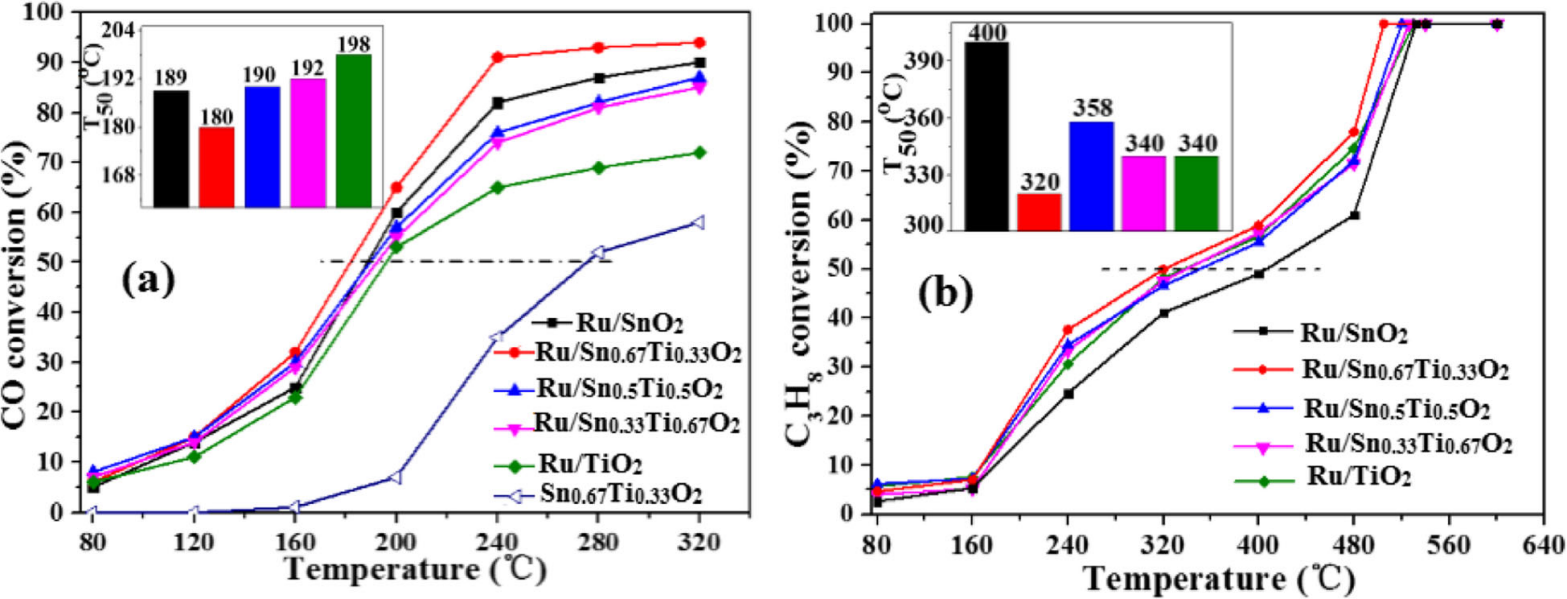
Effect of different Sn/Ti molar ratios on Ru/Sn_*x*_Ti_1−*x*_O_2_ catalytic oxidation of CO (**a**) and C_3_H_8_ (**b**)

**Fig. 2:**
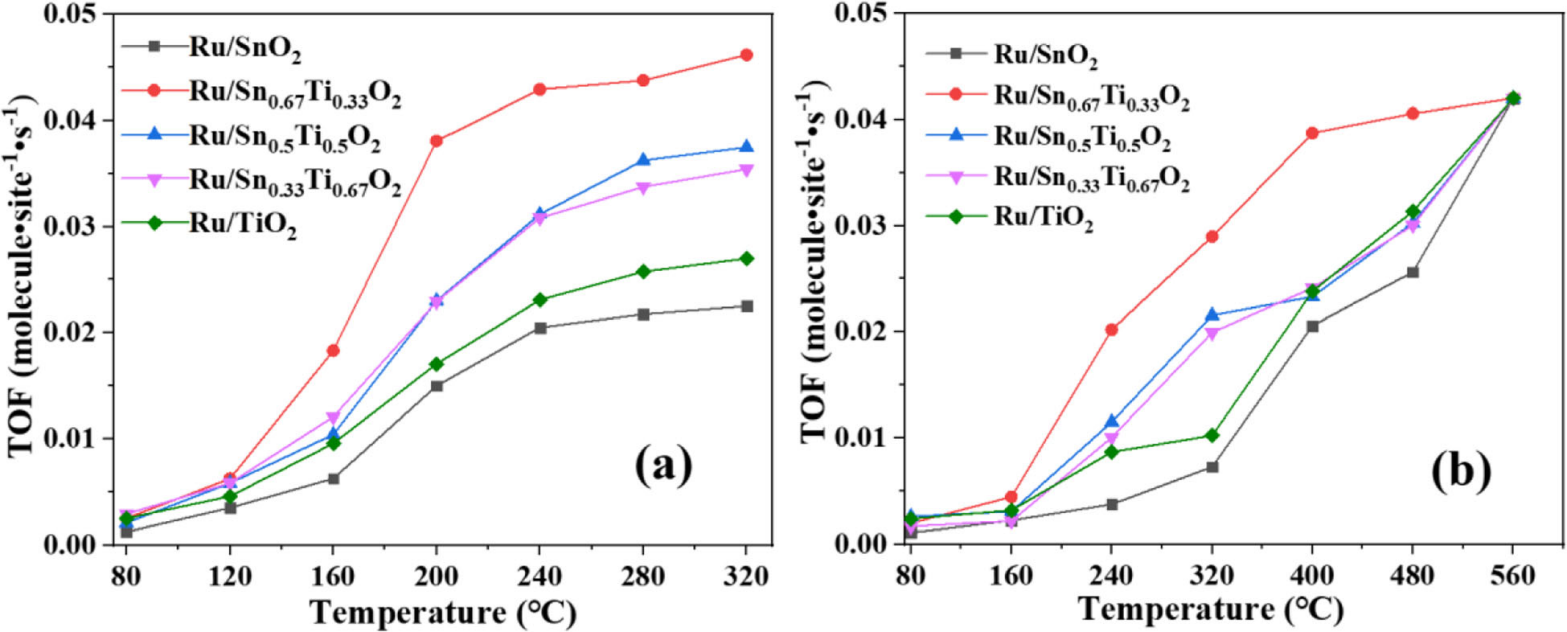
TOF of reaction temperature for CO (**a**) and C_3_H_8_ (**b**) oxidation over various catalysts

Figure 1 shows the catalytic activities of CO and C_3_H_8_ oxidation on the Ru/Sn_*x*_Ti_1−*x*_O_2_ catalysts under the optimal preparation conditions (Fig. S1, S2 and S3) of hydrothermal temperature at 180 °C, hydrothermal time at 24 h, and calcination temperature at 400 °C. It can be seen that catalytic performances of Ru/Sn_*x*_Ti_1−*x*_O_2_ catalysts increased firstly and then tended to be stabilized with the increase of reaction temperature. When the molar ratio of Sn/Ti is 2/1, the *T*_50_ of Ru/Sn_0.67_Ti_0.33_O_2_ to oxidize CO and C_3_H_8_ is 180 °C and 320 °C, respectively, which is lower reaction temperature than other Sn/Ti molar ratios. The conversion of CO reached 90% at 240 °C, and the complete conversion of C_3_H_8_ could be achieved at 500 °C on the Ru/Sn_0.67_Ti_0.33_O_2_ catalyst. The catalytic performance for each sample was normalized with respect to the Ru atoms on the surface and expressed in terms of turnover frequency (TOF), as shown in Fig. 2. The TOF value for Ru/Sn_0.67_Ti_0.33_O_2_ was the highest among all the samples at any reaction temperature. It is attributed to the highly dispersed Ru on the surface of Sn_0.67_Ti_0.33_O_2_, and the active component Ru has a strong interaction with the carrier Sn_0.67_Ti_0.33_O_2_ [22, 26]. Sharif et al. [6] reported that the conversion of Ru/[Ca_24_Al_28_O_64_]^4+^(O^2−^)_2_ to CO was only 82% at 240 °C. Murayama et al. [27] reported that the conversion of Au/Nb_2_O_5_ and Au/SiO_2_ to CO was 55% and 38%, respectively, at 250 °C. Compared with other literatures [27, 28], when the molar ratio of Sn/Ti is 2/1, higher CO conversion can be achieved at lower temperature in this study. Okal et al. [29] reported that the *T*_50_ of CH_4_ oxidized by Ru/ZnAl_2_O_4_ catalysts was 480, 500, and 540 °C, when the loading of Ru was 0.5 wt.%, 1.0 wt.%, and 4.5 wt.%, respectively. Wilburn et al. [30] reported that the *T*_50_ of CH_4_ oxidation over 0.3Pd–0.7Pt/γ–Al_2_O_3_ catalyst was 360 °C. The catalytic activities of different catalysts for CO and C_3_H_8_ oxidation are shown in Table S1 and Table S2. Complete transformation of C_3_H_8_ can be achieved at a lower temperature in this work. The optimum molar ratio of Sn/Ti is 2/1, which is consistent with the activity of CO. From the above analysis, it can be concluded that the conversion of CO and C_3_H_8_ is greatly affected by the molar ratio of Sn/Ti. When the molar ratio of Sn/Ti is 2/1, the *T*_50_ of Ru/Sn_0.67_Ti_0.33_O_2_ to CO and C_3_H_8_ is 180 °C and 320 °C, respectively. When the reaction temperature is 240 °C, the conversion of CO can reach 90% and the complete conversion of C_3_H_8_ can be achieved when the reaction temperature is 500 °C.

**Fig. 3:**
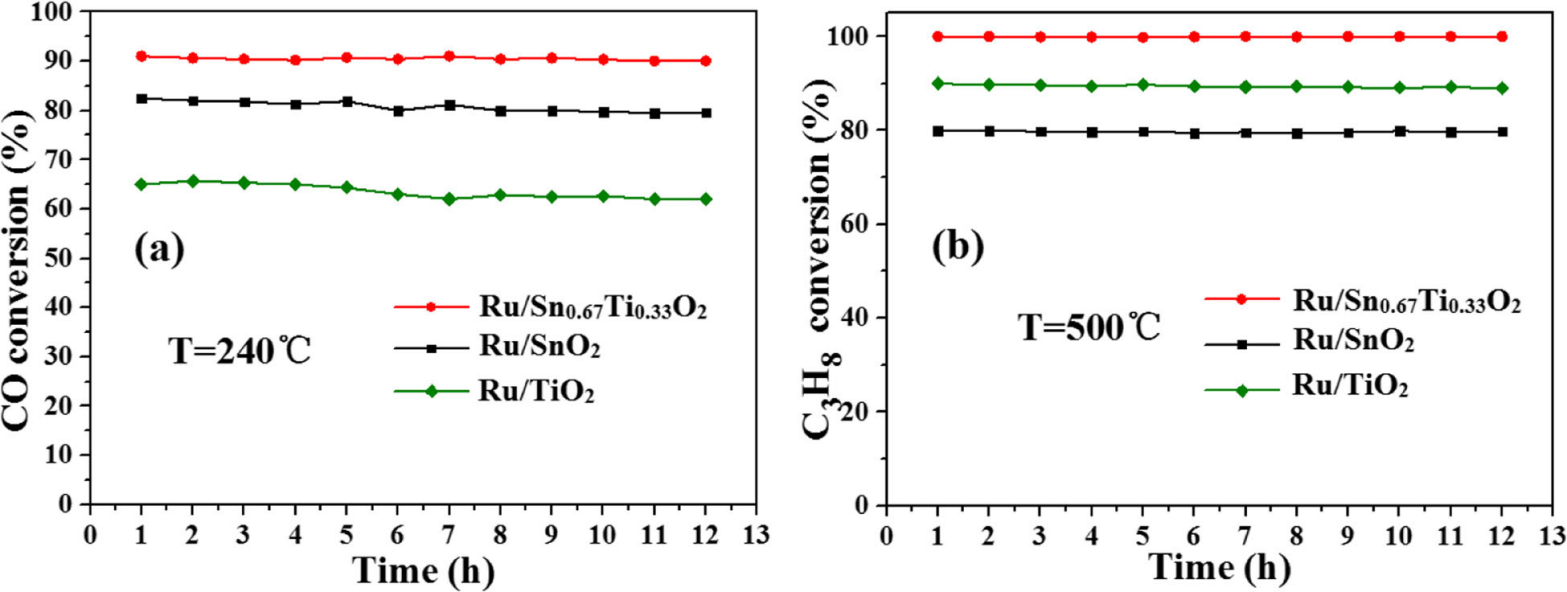
The stability of Ru/Sn_*x*_Ti_1−*x*_O_2_ catalytic CO (**a**) and C_3_H_8_ (**b**)

The stability of CO and C_3_H_8_ was investigated in Fig. 3, under hydrothermal temperature at 180 °C, hydrothermal time at 24 h, and calcination temperature at 400 °C (Fig. S1, S2 and S3). The conversion of CO reached 90% at 240 °C, and the complete conversion of C_3_H_8_ could be achieved at 500 °C. Interestingly, Ru/Sn_0.67_Ti_0.33_O_2_ catalyst is basically inactivated after a 12 h catalytic reaction; however, the activity of Ru/TiO_2_ and Ru/SnO_2_ catalysts decreased slightly with the increase of time when they oxidized CO. The phenomenon indicates that the formation of Sn_*x*_Ti_1−*x*_O_2_ solid solution can not only improve the activity of the catalysts, but also increase the stability. It is attributed that Ru is highly dispersed on the surface of Sn_0.67_Ti_0.33_O_2_; there is a strong interaction between the active component Ru and the carrier Sn_0.67_Ti_0.33_O_2_ [26].

### Catalyst Characterization

#### Texture Properties of Sn_*x*_Ti_1−*x*_O_2_ Supports and Ru/Sn_x_Ti_1−*x*_O_2_ Catalysts

**Fig. 4:**
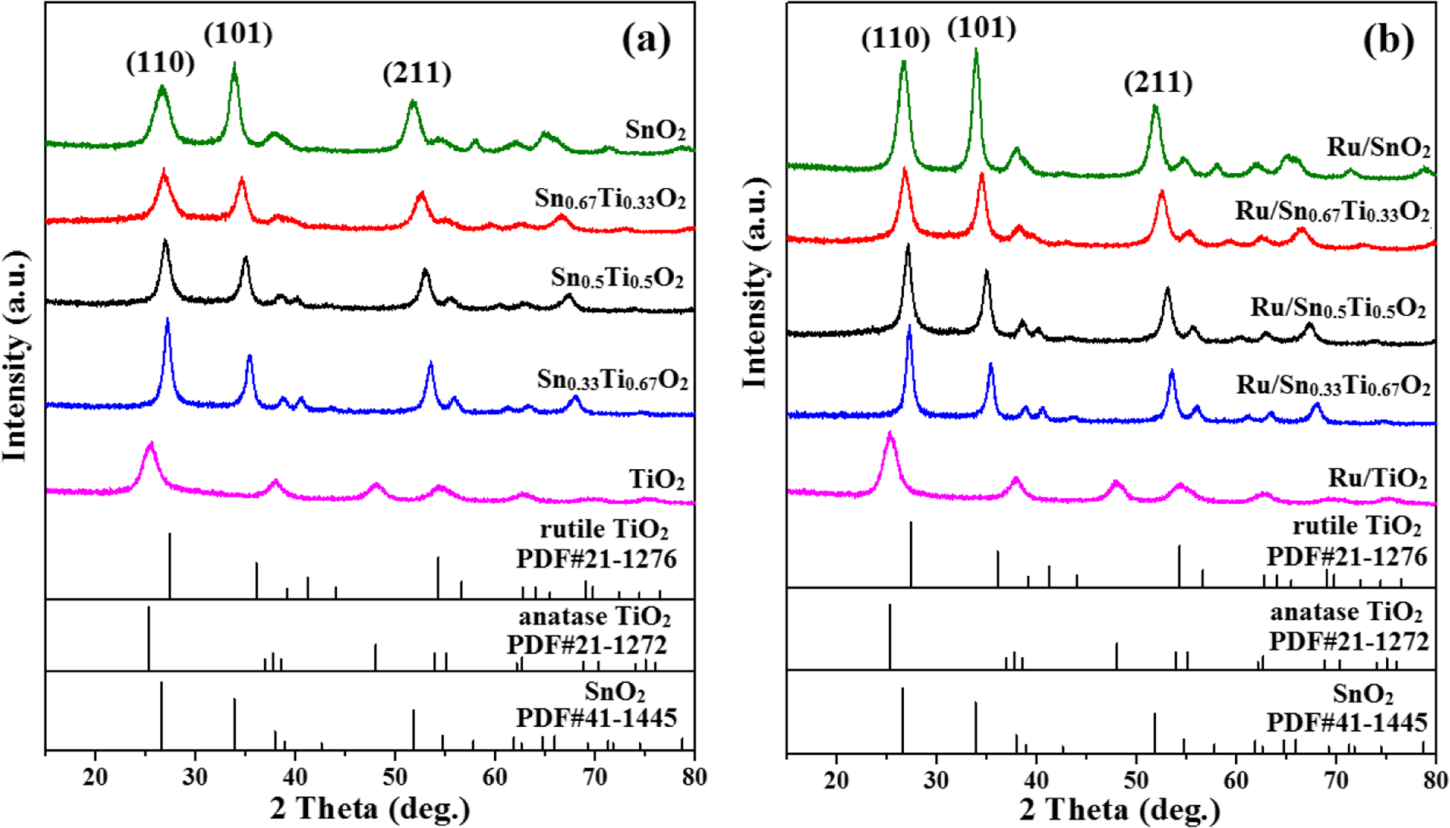
XRD patterns of Sn_*x*_Ti_1−*x*_O_2_ supports (**a**) and Ru/Sn_*x*_Ti_1−*x*_O_2_ catalysts (**b**)

Figure 4 shows the XRD patterns of both Sn_*x*_Ti_1−*x*_O_2_ solid solution and Ru/Sn_*x*_Ti_1−*x*_O_2_ catalysts. Typical peaks of anatase structure are observed in the TiO_2_ (25.78°) and Ru/TiO_2_ (25.67°) samples with grain sizes of about 4 nm and 5.5 nm (Table 1), respectively. A phase transition from anatase to rutile appeared with the introduction of Sn. The Ru diffraction peaks are not observed, indicating that Ru is highly dispersed on Sn_*x*_Ti_1−*x*_O_2_ surface or beyond the XRD detection limitation [31]. Furthermore, the diffraction peaks of Sn_*x*_Ti_1−*x*_O_2_ and Ru/Sn_*x*_Ti_1−*x*_O_2_ move gradually to lower angles with increasing Sn content, suggesting that the interplanar spacing *d* increases according to the Bragg equation, 2*d* sin*θ* = *nλ*. This is consistent with the increase in tetragonal lattice parameters (*a* and *c*) in Table 1, which is attributed to the substitution of larger ionic radius Sn^4+^ (0.071 nm) for Ti^4+^ (0.068 nm). The results suggest the Sn^4+^ has been successfully doped into the TiO_2_ lattice to form a uniform (–Sn^4+^–O–Ti^4+^–) solid solution while maintaining the rutile phase structure, which is in agreement with some previous studies [5, 18].

**Table 1:** Texture properties of Sn_*x*_Ti_1−*x*_O_2_ supports and Ru/Sn_*x*_Ti_1−*x*_O_2_ catalysts

Samples	Crystalline size (nm)	Lattice parameter *a* = *b*, *c* (nm)	BET surface area (m^2^ g^−1^)	Vp/cm^3^ g^−1^	*d*_*p*_/*A*
SnO_2_	6	*a* = 4.738, *c* = 3.187	126.4	0.14	4.0
Sn_0.67_Ti_0.33_O_2_	4.5	*a* = 4.680, *c* = 3.095	156.5	0.17	3.9
Sn_0.5_Ti_0.5_O_2_	5	*a* = 4.666, *c* = 3.073	126.2	0.18	5.0
Sn_0.33_Ti_0.67_O_2_	5.4	*a* = 4.637, *c* = 3.027	108.1	0.20	6.5
TiO_2_	4	*a* = 3.784, *c* = 2.959	257.4	0.21	3.5
Ru/SnO_2_	23	*a* = 4.738, *c* = 3.187	77.34	0.14	6.1
Ru/Sn_0.67_Ti_0.33_O_2_	7.6	*a* = 4.709, *c* = 3.141	83.3	0.15	5.3
Ru/Sn_0.5_Ti_0.5_O_2_	9.5	*a* = 4.666, *c* = 3.073	75.3	0.15	5.6
Ru/Sn_0.33_Ti_0.67_O_2_	11	*a* = 4.643, *c* = 3.044	68.6	0.16	6.5
Ru/TiO_2_	5.5	*a* = 3.785, *c* = 2.961	177.10	0.23	5.3

**Fig. 5:**
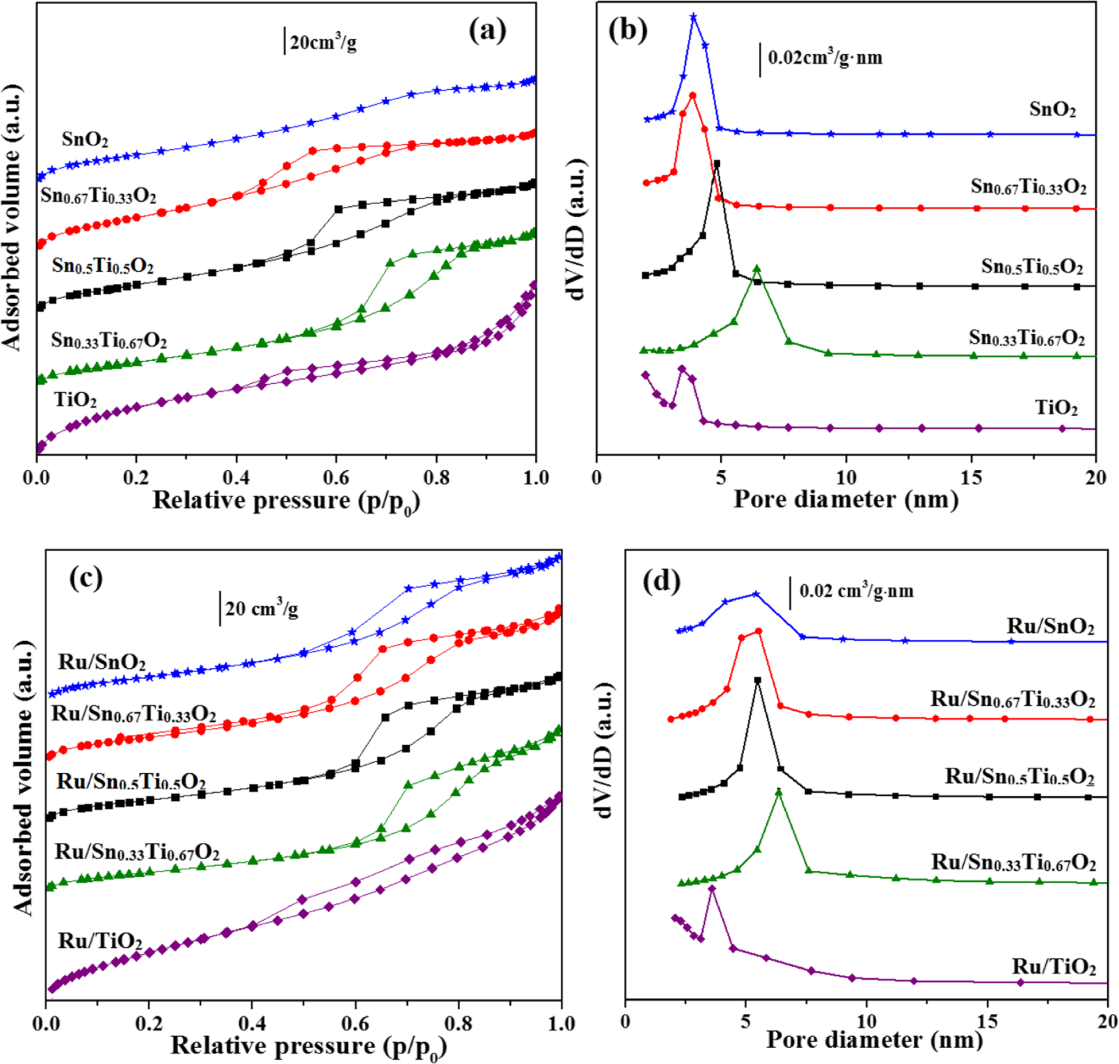
N_2_ adsorption-desorption isotherms of Sn_*x*_Ti_1−*x*_O_2_ (**a**) and Ru/Sn_*x*_Ti_1−*x*_O_2_ (**c**) the pore size distribution of Sn_x_Ti_1−*x*_O_2_ (**b**) and Ru/Sn_*x*_Ti_1−*x*_O_2_ (**d**)

To determine the texture properties of samples, the N_2_ adsorption-desorption technique was used. The N_2_ adsorption-desorption isotherms and corresponding pore diameter distribution curves of these samples are shown in Fig. 5. The N_2_ adsorption-desorption isotherms of SnO_2_ distinctly belong to type II; others are classical type IV according to IUPAC classification and present a H2 complex hysteresis loop in a *p/p*_*0*_ range of 0.4–0.95, which is a common feature of mesoporous material (Fig. 5a, c) [17, 32]. The existence of these mesopores is an important reason for the large specific surface area of catalysts [33]. All of Sn_*x*_Ti_1−*x*_O_2_ supports and Ru/Sn_*x*_Ti_1−*x*_O_2_ catalysts exhibited a narrow distribution of small-sized pores (3–8 nm), especially the Sn_0.67_Ti_0.33_O_2_ support and Ru/Sn_0.67_Ti_0.33_O_2_ catalysts, with the pore diameter mainly uniformly distributed around 5 nm (Fig. 5b, d). This phenomenon suggested that an appropriate amount of Sn can weaken the diffusion coefficient of the catalytic surface and indirectly hinder the agglomeration of the crystallites [17].

The texture properties of Sn_*x*_Ti_1−*x*_O_2_ supports and Ru/Sn_*x*_Ti_1−*x*_O_2_ catalysts are listed in Table 1. The specific surface area and pore distribution were calculated by the BET and BJH method. Both the specific surface area and pore volume of Sn_0.67_Ti_0.33_O_2_ are 156.5 m^2^ g^−1^ and 0.17 cm^3^ g^−1^, respectively. But both specific surface area and pore volume of the Ru/Sn_0.67_Ti_0.33_O_2_ catalyst are decreased compared with the Sn_0.67_Ti_0.33_O_2_ support, which indicates that Ru loaded on the Sn_0.67_Ti_0.33_O_2_ surface. Moreover, the Ru/Sn_0.67_Ti_0.33_O_2_ catalyst is sintered and the open pore structure collapsed to form plugged pores during the high-temperature calcination process [31]. Nevertheless, Ru/Sn_0.67_Ti_0.33_O_2_ still maintains larger specific surface area (83.3 m^2^ g^−1^) and smaller pore diameter (5.3 nm) in comparison with other rutile samples such as Ru/Sn_0.33_Ti_0.67_O_2_, Ru/Sn_0.5_Ti_0.5_O_2_, and Ru/SnO_2_.

**Fig. 6:**
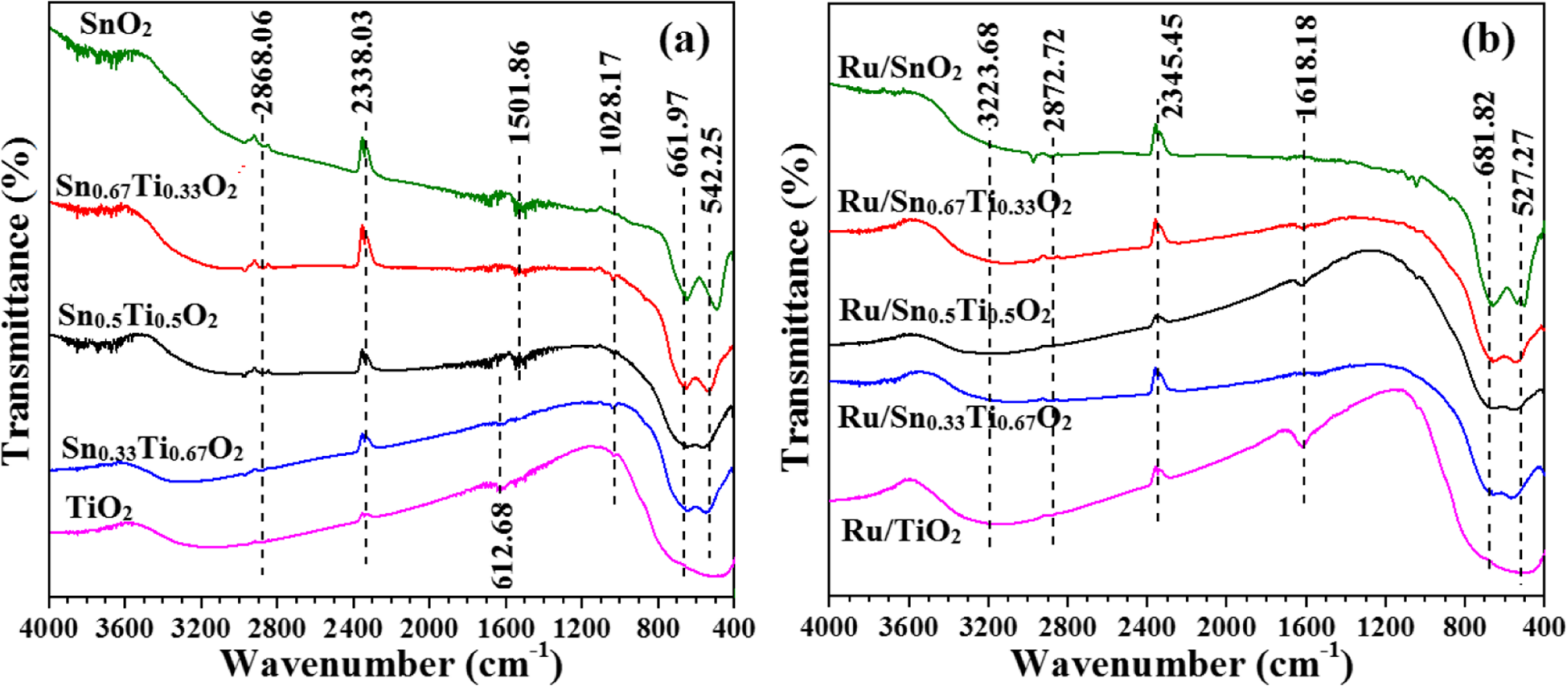
FT-IR spectra of the Sn_*x*_Ti_1−*x*_O_2_ supports (**a**) and Ru/Sn_*x*_Ti_1−*x*_O_2_ catalysts (**b**)

Figure 6 shows the FT-IR spectra of Sn_*x*_Ti_1−*x*_O_2_ supports and Ru/Sn_*x*_Ti_1−*x*_O_2_ catalysts. All the samples present similar vibration peaks at analogous wavenumber positions. The adsorption at around 3223.68 cm^−1^ is due to surface hydroxyl groups neighboring oxygen vacancy sites [34, 35]. The bands of 1501.86–1618.18 cm^−1^ belong to the angular vibration peak of water. The symmetrical stretching vibration peak of lattice oxygen appears at 1028.17 cm^−1^. The band of 527.27–681.2 cm^−1^ may be attributed to the stretching vibration peak of TiO_2_ or SnO_2_ [34]. Compared with Sn_*x*_Ti_1−*x*_O_2_ supports, Ru/Sn_*x*_Ti_1−*x*_O_2_ spectrum broadens, indicating that the active component Ru and support Sn_*x*_Ti_1−*x*_O_2_ have some interaction, resulting in the surface defects of catalysts [36, 37].

### Morphology of Catalysts

**Fig. 7:**
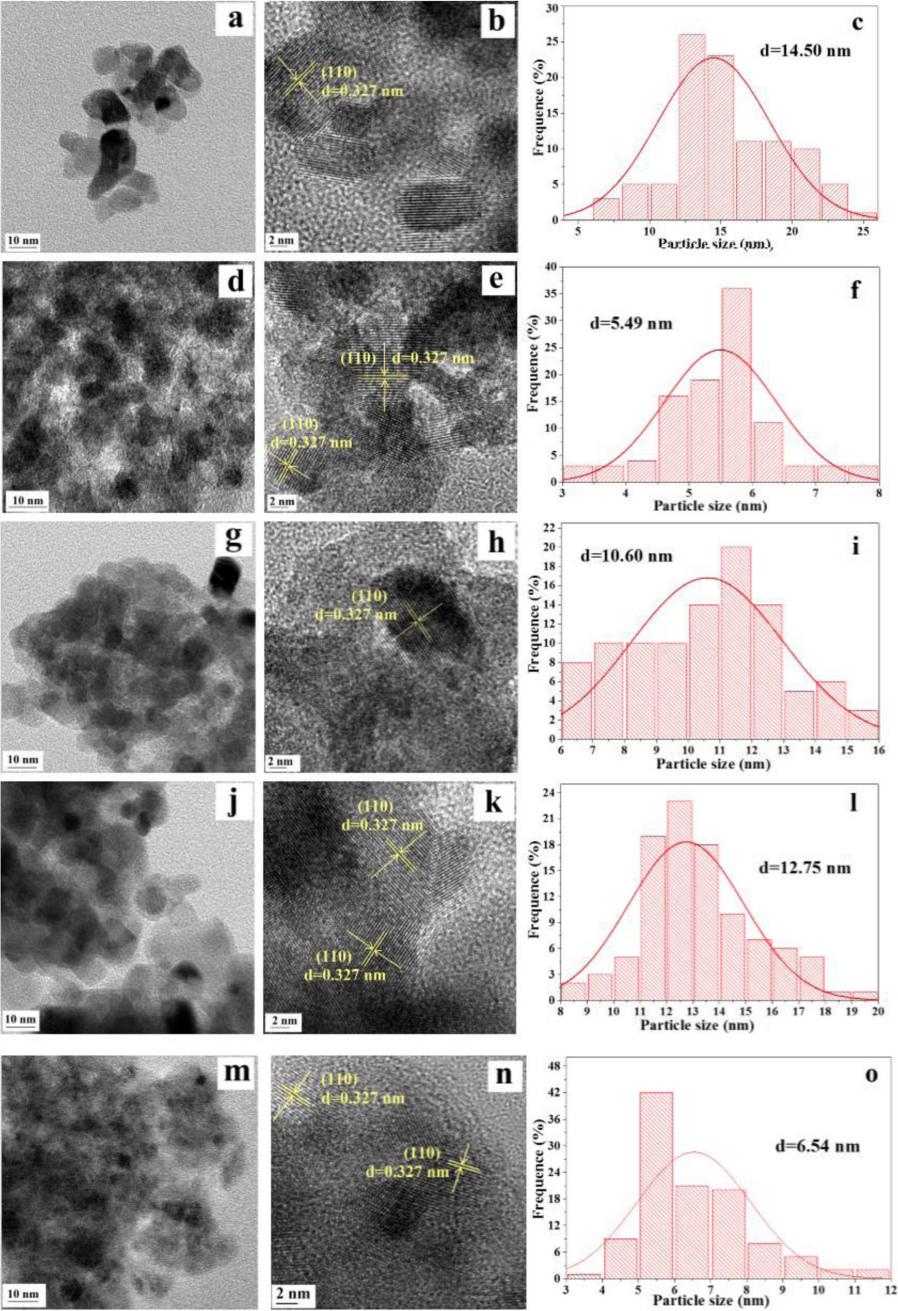
TEM, HRTEM images, and the particle size distribution of **a**, **b**, **c** Ru/SnO_2_; **d**, **e**, **f** Ru/Sn_0.67_Ti_0.33_O_2_; **g**, **h**, **i** Ru/Sn_0.5_Ti_0.5_O_2_; **j**, **k**, **l** Ru/Sn_0.33_Ti_0.67_O_2_; and **m**, **n**, **o** Ru/TiO_2_

Low- and high-resolution TEM, HRTEM images, and the particle size distribution of Ru/Sn_*x*_Ti_1−*x*_O_2_ are exhibited in Fig. 7. Based on the observation of the TEM images presented in Fig. 7a, d, g, j, and m, we find that all samples are composed of well-defined particles with irregular shapes and disordered mesoporous structure, which is formed by the agglomeration of the nanoparticles [38]. Furthermore, it can be seen that the Ru/Sn_0.67_Ti_0.33_O_2_ sample has the highest degree of agglomeration because of the smallest grain size among these samples. From the HRTEM images (Fig. 7b, e, h, k, n), there is only one kind of lattice fringes with 0.327 nm, which is compatible with (110) plane of these samples. Besides, we find that the lattice fringes of TiO_2_ and SnO_2_ are not observed, which is attributed to Sn^4+^ having been successfully doped into the lattice of TiO_2_ to form a homogeneous Sn_*x*_Ti_1−*x*_O_2_ solid solution [39]. The results are consistent with XRD. The Ru particle size distribution (Fig. 7c, f, i, l, o) shows that the approximate sizes of Ru particles ranged from 3 to 20 nm. The introduction of Sn^4+^ could effectively decrease the sizes of Ru particles and achieve a higher dispersion on the Sn_*x*_Ti_1−*x*_O_2_ surface. Comparing with other samples, the Ru particle size distribution of Ru/Sn_0.5_Ti_0.5_O_2_ sample was wider (< 13 nm), which may be caused by the interaction between (–Sn^4+^–O–Ti^4+^–) species and Ru [26]. The Ru/Sn_0.67_Ti_0.33_O_2_ catalyst has better Ru dispersion and smaller particle size (5.49 nm) among all samples.

### Surface Properties of Catalysts

**Fig. 8:**
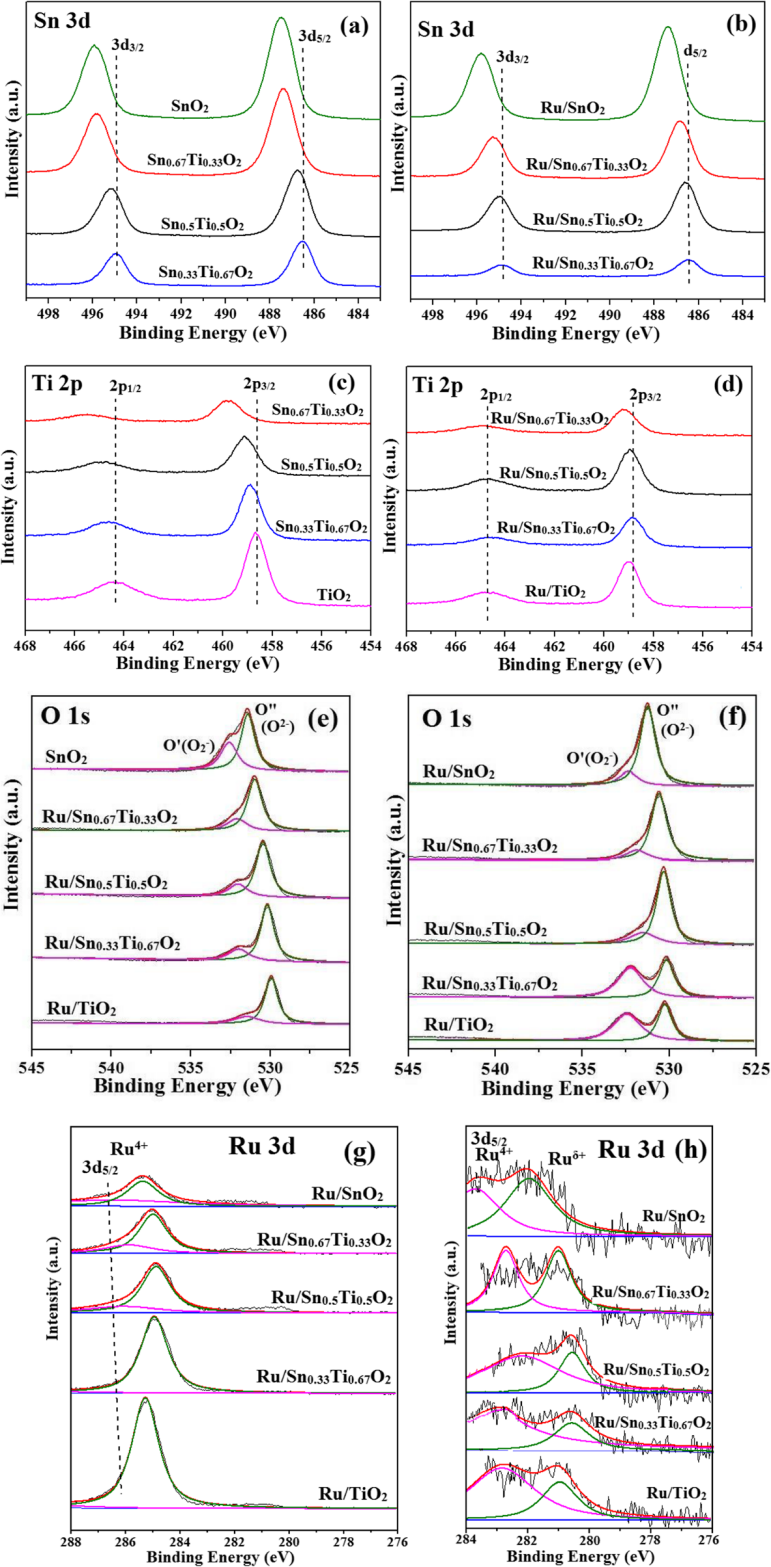
XPS spectra (Sn3d (**a**, **b**), Ti2p (**c**, **d**), O1s (**e**, **f**) and Ru3d (**g**, **h**)) of Sn_*x*_Ti_1−*x*_O_2_ supports and Ru/Sn_*x*_Ti_1−*x*_O_2_ catalysts

To further determine the elementary states and surface composition, XPS analysis was carried out. Figure 8 shows the XPS spectra of Sn 3d, Ti 2p, O 1s, and Ru 3d for the Sn_*x*_Ti_1−*x*_O_2_ supports and Ru/Sn_*x*_Ti_1−*x*_O_2_ catalysts. The XPS binding energy values of the Sn 3d_3/2_ and Sn 3d_5/2_ are observed at 486.6–487.5 eV and 494.9–496.1 eV, respectively, which are characteristic of Sn^4+^ species in Sn_*x*_Ti_1−*x*_O_2_ supports or Ru/Sn_*x*_Ti_1−*x*_O_2_ catalysts. Interestingly, the binding energy of Sn 3d_3/2_ and Sn 3d_5/2_ shifted to higher values after the introduction of Sn^4+^, indicating some of the Sn^4+^ replace the Ti^4+^ sites and have a strong interaction with TiO_2_, which is in agreement with XRD. Also, the oxyen vacancies may be created by the lower valent Sn^δ+^ [5]. Two peaks corresponding to Ti 2p_3/2_ and Ti 2p_1/2_ are observed at 458.7–459.9 eV and 464.3–465.8 eV in the XPS spectra of Ti 2p, suggesting that Ti^4+^ and Ti^3+^ existed in the samples, and the binding energy values of Ti 2p_3/2_ and Ti 2p_1/2_ shifted to higher binding energy values with the increase of Sn^4+^, further proving the existence of oxygen vacancies. It can be seen from Table 3 that the Sn/Ti molar ratio by XPS is observed to be slightly higher than theoretical calculation, indicating that Sn is enriched on the surface of catalysts, which leads to more oxygen vacancies. Because the electronegativity of Sn (1.96) is larger than that of Ti (1.62), in other words, the electron-capturing ability of Sn is stronger than that of Ti, which causes the redox equilibrium (Sn^4+^+Ti^3+^ → Sn^δ+^+Ti^4+^) shifting to right [32].

The high-resolution spectra of the O 1s ionization feature are numerically consistent with the Gaussian feature and deconvoluted into two peaks [5]. Higher binding energy (O’, 532.1 eV) is caused by chemisorbed oxygen that formed the (O_2_^−^, O^−^, or O_2_^2−^) species. However, the O” (529.9 eV) is the characteristic peak of O^2−^ on the surface of metal oxides. Interestingly, the binding energy of O 1s shifted to higher values after the addition of Sn^4+^.

Ru 3d spectra present Ru^4+^ and lower value Ru^δ+^. The signal of Ru 3d_5/2_ is often used to analyze the charge state of the Ru species, since another Ru 3d_3/2_ overlaps with C 1s at around 284.0 eV [40]. The binding energy of 282.0–283.5 eV is assigned to Ru 3d_5/2_, which corresponded to Ru^4+^. The lower binding energy at around 280.2–281.7 eV is attributed to lower state Ru^δ+^, and the Ru^δ+^ relative ratio in Ru/Sn_0.67_Ti_0.33_O_2_ reaches 53.9%, which is higher than other catalysts. It could be explained that the strong interaction between Sn_0.67_Ti_0.33_O_2_ and Ru caused a larger amount of surface reactive oxygen species [26].

XPS and EDS analyses are performed to determine the surface and bulk composition of the samples as shown in Table 2. Surface and bulk Ru analysis shows that Ru/Sn_0.67_Ti_0.33_O_2_ has the highest surface Ru (0.69 wt.%) and bulk Ru (0.40 wt.%) among all the catalysts, indicating that the active component Ru is more evenly distributed on the Sn_0.67_Ti_0.33_O_2_ support, and more Ru species enters the internal of Sn_0.67_Ti_0.33_O_2_ to form a strong interaction.

**Table 2:** The surface compositions by XPS and bulk compositions by EDS of the Sn_*x*_Ti_1−*x*_O_2_ supports and Ru/Sn_*x*_Ti_1−*x*_O_2_ catalysts

Samples	C 1s	O 1s	Ti 2p	Sn 3d	Sn/Ti	Ru (XPS)	Ru (EDS)
SnO_2_	21.4	62.06	0	16.90			
Sn_0.67_Ti_0.33_O_2_	31.23	48.35	5.98	14.44	2.41		
Sn_0.5_Ti_0.5_O_2_	22.51	56.16	9.57	11.75	1.23		
Sn_0.33_Ti_0.67_O_2_	23.41	55.70	13.20	7.69	0.58		
TiO_2_	36.59	44.00	19.41	0	0		
Ru/SnO_2_	16.35	58.58	0	24.94		0.37	0.20
Ru/Sn_0.67_Ti_0.33_O_2_	22.95	53.58	7.66	15.62	2.04	0.69	0.40
Ru/Sn_0.5_Ti_0.5_O_2_	22.4	53.82	11.33	12.27	1.08	0.64	0.28
Ru/Sn_0.33_Ti_0.67_O_2_	29.88	56.95	8.33	4.56	0.55	0.41	0.26
Ru/TiO_2_	37.23	50.89	11.50	0	0	0.38	0.24

**Fig. 9:**
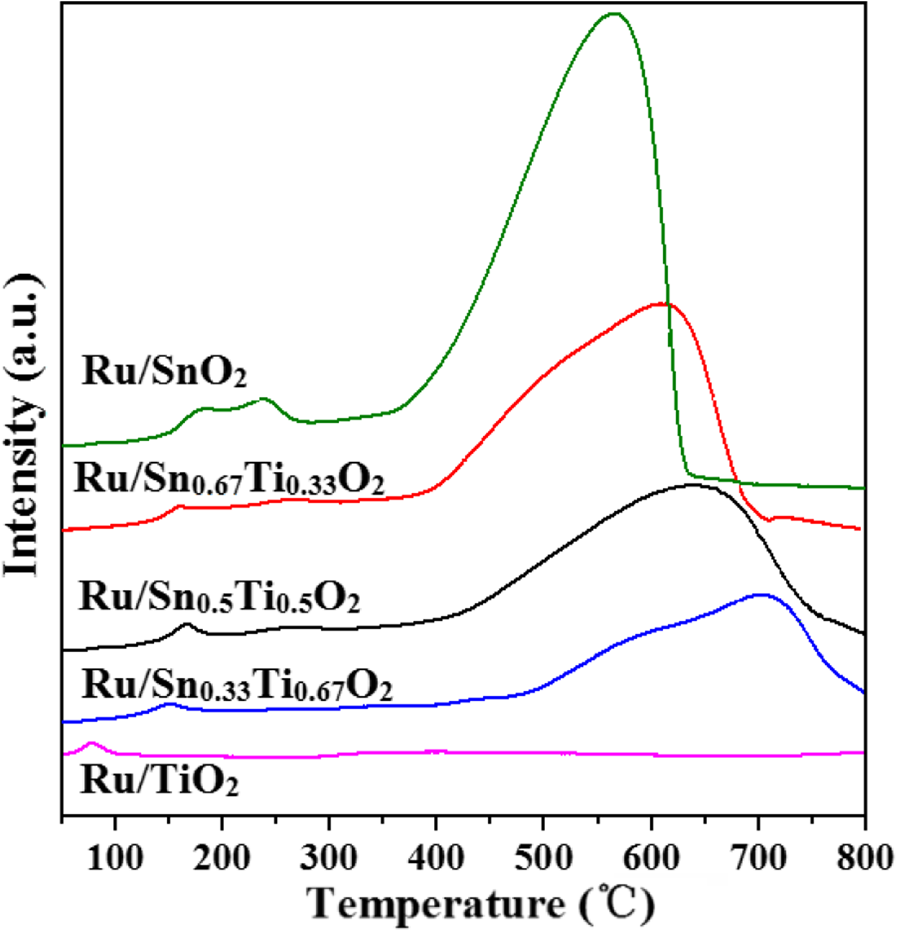
H_2_-TPR spectra of Ru/Sn_*x*_Ti_1−*x*_O_2_ catalysts

In order to further investigate the reduction performance of the Ru/Sn_*x*_Ti_1−*x*_O_2_ catalysts, temperature-programmed reduction studies are performed (Fig. 9). The shapes of these H_2_-TPR profiles are almost identical. The reduction peaks of Ru/Sn_*x*_Ti_1−*x*_O_2_ are divided into two parts: the low-temperature reduction peaks 80–270 °C are associated to the lower state Ru^δ+^ reduced from RuO_2_ and a significant amount of Sn^4+^ which could be reduced to lower valent Sn^δ+^ or can be attributed to the reduction of surface oxygen [41], while the high-temperature reduction peaks 600–640 °C are associated to Sn^0^ reduced from Sn^δ+^ or the reduction of bulk oxygen of catalysts [26, 42], which is consistent with XPS results. The reduction temperature of Ru/Sn_*x*_Ti_1−*x*_O_2_ moves towards lower temperature, peaks broaden and H_2_ consumption increase with the addition of Sn, and hydrogen consumption from the H_2_-TPR measurements are shown in Table 3. The dispersion of active components on the surface of the samples has a significant effect on the reduction of surface oxygen, and hydrogen could be more easily activated with higher dispersion of Pd, resulting in the increase of H_2_ consumption [43]. Therefore, we can infer that the introduction of Sn significantly increased the dispersion of Ru on the carrier, which may have resulted from the formation of Sn_*x*_Ti_1−*x*_O_2_ solid solution. The results are in good agreement with XRD and TEM. Because the reduction of TiO_2_ is usually difficult to conduct at low temperature, there are no peaks of the TiO_2_ reduction observed during the H_2_-TPR from 50 to 800 °C [15]. Nevertheless, the Ru/Sn_0.67_Ti_0.33_O_2_ still exhibits a higher H_2_ consumption.

**Table 3:** Hydrogen consumption of Ru/Sn_*x*_Ti_1−*x*_O_2_ catalysts

Samples	Total H_2_ consumption (μmol/g)	H_2_ consumption at 80–270 °C (μmol/g)	H_2_ consumption at 400–640 °C (μmol/g)
Ru/SnO_2_	11694.59	854.18	10840.41
Ru/Sn_0.67_Ti_0.33_O_2_	8273.80	749.44	7524.36
Ru/Sn_0.5_Ti_0.5_O_2_	7001.22	563.05	6438.17
Ru/Sn_0.33_Ti_0.67_O_2_	5699.90	432.77	5267.13
Ru/TiO_2_	474.80	396.63	78.17

**Fig. 10:**
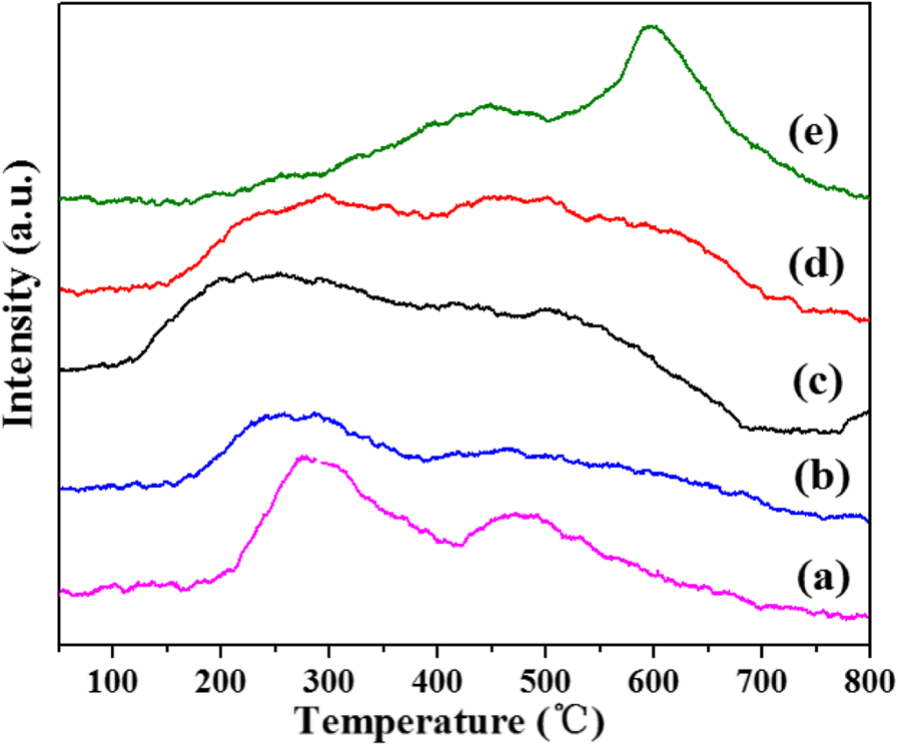
O_2_-TPD spectra of Ru/TiO_2_ (**a**), Ru/Sn_0.33_Ti_0.67_O_2_ (**b**), Ru/Sn_0.5_Ti_0.5_O_2_ (**c**), Ru/Sn_0.67_Ti_0.33_O_2_ (**d**), and Ru/SnO_2_ catalysis

The O_2_-TPD experiments (Fig. 10) of Ru/Sn_*x*_Ti_1−*x*_O_2_ samples are imposed to gain insight into the mobility of surface and lattice oxygen. The signal at low temperature (< 200 °C) is attracted by the desorption of surface chemisorbed oxygen (O_2_^−^, O_2_^2−^, or O^−^ species); the main peak centered at 280 °C or 500 °C which is attributed to the desorption of the structure oxygen species, and the peaks above 600 °C are assignable to the desorption of the lattice oxygen (O^2−^) species [44]. The incorporation of Sn increased the adsorbed oxygen species and shifted to a lower temperature [45]. The results indicate that the incorporation of Sn improved the oxygen activation ability of the Ru/Sn_*x*_Ti_1−*x*_O_2_ samples and the interaction between the carriers Sn_*x*_Ti_1−*x*_O_2_ and active component Ru [46, 47].

### CO and/or O_2_ Interaction with these Samples

**Fig. 11:**
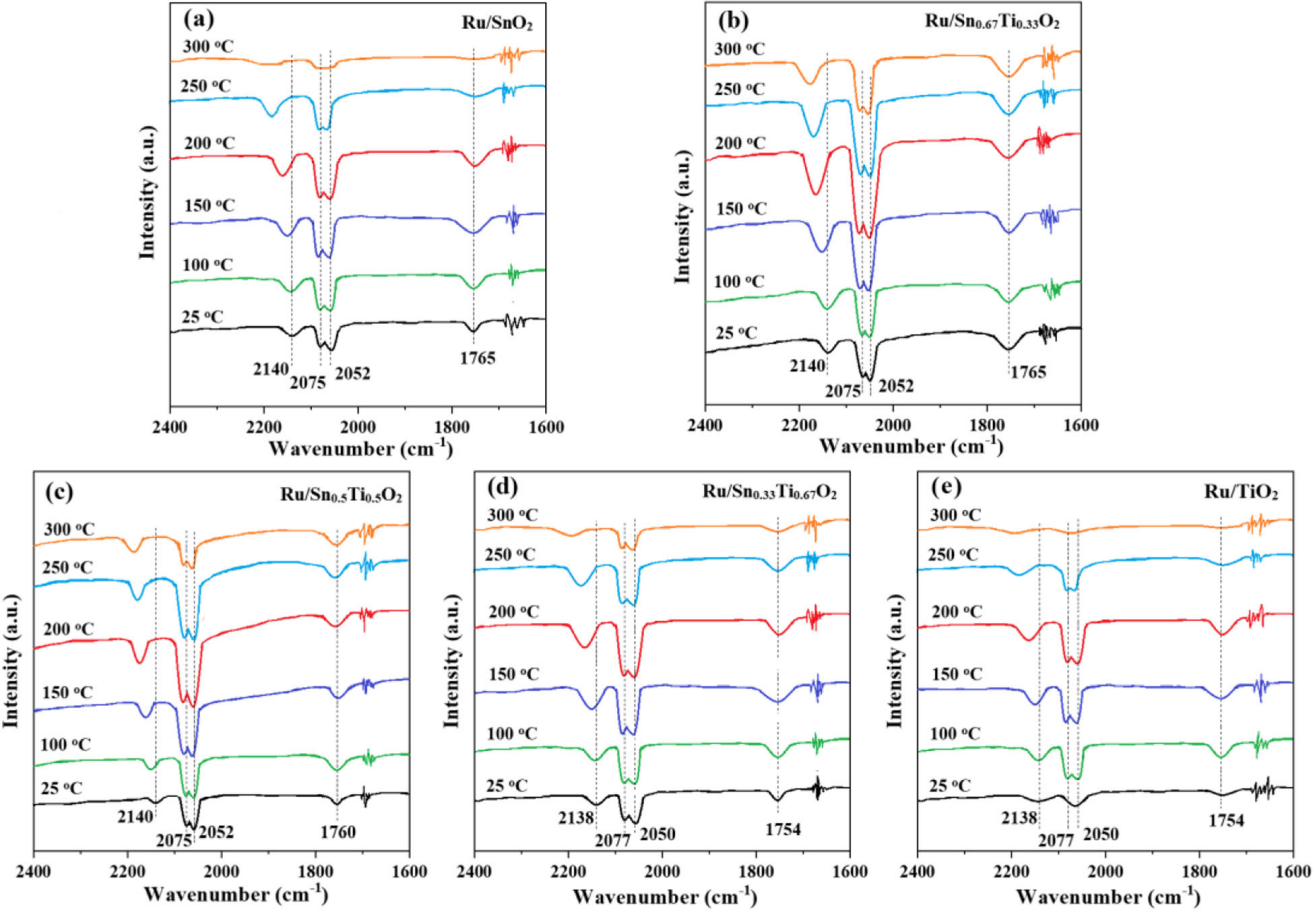
In situ FI-IR spectra of the 10% CO/Ar interaction with **a** Ru/SnO_2_, **b** Ru/Sn_0.67_Ti_0.33_O_2_, **c** Ru/Sn_0.5_Ti_0.5_O_2_, **d** Ru/Sn_0.33_Ti_0.67_O_2_, **e** Ru/TiO_2_ at different temperatures

The in situ FI-IR spectra of CO adsorption are recorded to further investigate the effect of the ruthenium oxide species, as shown in Fig. 11. The band located at 2052 cm^−1^ is attributed to linear CO adsorbed on reduced Ru crystallites (Ru^δ+^–CO), the band at 2140 cm^−1^ and 2075 cm^−1^ can be assigned to two different types of multicarbonyl species on partially oxidized Ru sites (Ru^n+^(CO)_*x*_), and the band at 1765 cm^−1^ is attributed to (Sn_*x*_Ti_1−*x*_O_2_)Ru–CO species [48, 49]. The Ru^δ+^–CO adsorption peaks at room temperature indicate the presence of some lower state Ru^δ+^ species. This is in agreement with the XPS results. However, the desorption temperature of the Ru^δ+^–CO peak is related to the Sn/Ti ratio and temperature. As the temperature increases, the peak intensity enhances firstly and then decreases gradually. Simultaneously, the CO adsorption peak moves to a higher wave number (2052 cm^−1^ at 25 °C and 2060 cm^−1^ at higher temperatures). This red-shift indicates that Sn^4+^ has stronger electron-donating capability [50]. For the Ru/SnO_2_, Ru/Sn_0.5_Ti_0.5_O_2_, Ru/Sn_0.33_Ti_0.67_O_2_, and Ru/TiO_2_ samples, the CO maximum adsorption peak on Ru^δ+^ appears at about 200 °C and disappears basically at 300 °C. For the Ru/Sn_0.67_Ti_0.33_O_2_ sample, the CO maximum adsorption peak on Ru^δ+^ appears at about 200 °C, which can be observed clearly even at 300 °C. It can be concluded that Ru^δ+^ is much more stable in Ru/Sn_0.67_Ti_0.33_O_2_ sample, which can provide more CO adsorption sites than in the other samples.

### Possible Reaction Mechanism over the Ru/Sn_*x*_Ti_1−*x*_O_2_ Catalysts

**Fig. 12:**
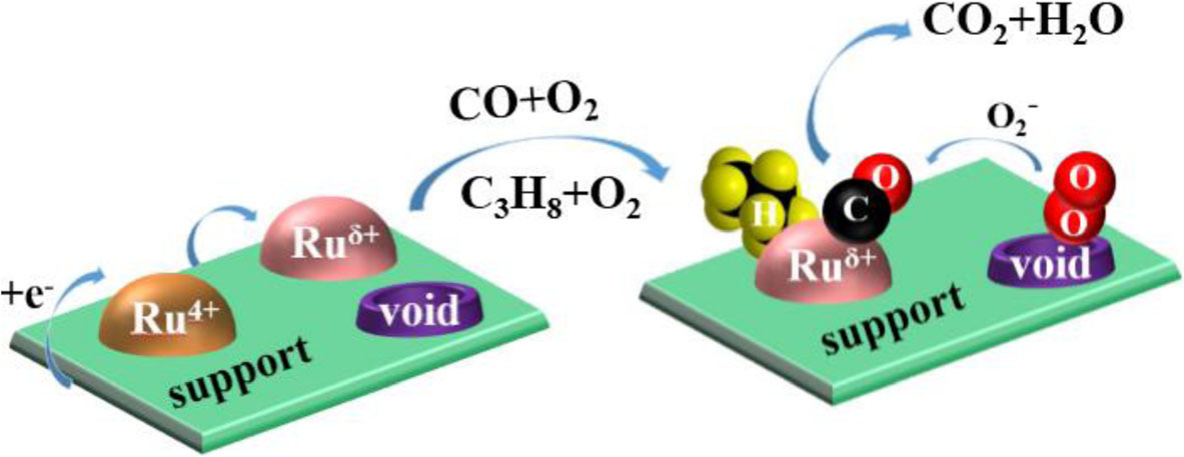
Possible reaction mechanism of CO and C_3_H_8_ over Ru/Sn_*x*_Ti_1−*x*_O_2_

According to the characterizations mentioned above, a possible reaction mechanism of CO and C_3_H_8_ oxidation is proposed and schematized in Fig. 12. Based on the XPS results, electrons migrate between Ru and Sn_*x*_Ti_1−*x*_O_2_ solid solution; because the electronegativity of Ru (2.22) is larger than that of Ti (1.62) and Sn (1.96), the electrons will transfer from the Sn_*x*_Ti_1−*x*_O_2_ solid solution to Ru^4+^, in which lower state Ru^δ+^ will be generated. Meanwhile, –Ti^4+^–O–Sn^4+^– species are oxidized and more oxygen will be absorbed on the surface of Sn_*x*_Ti_1−*x*_O_2_ solid solution, which can provide oxygen to the oxidation reaction of CO and C_3_H_8_. At the same time, the by-products produced in the oxidation process will also be adsorbed on the surface of Sn_*x*_Ti_1−*x*_O_2_ solid solution, which will not deteriorate the activity of Ru^δ+^ species. It is also the reason for the high stability of the catalysts. Moreover, the lower state Ru^δ+^ species have more metal properties, which play a crucial role in the activation of CO and C_3_H_8_ [40]. Compared with Ru/TiO_2_ and Ru/SnO_2_, high dispersion of Ru on Sn_*x*_Ti_1−*x*_O_2_ solid solution is also an important cause for their excellent activity and stability. Based on O_2_-TPD analysis, O_2_ is first adsorbed on the surface of catalysts to form O_2_^−^ species and CO and C_3_H_8_ adsorbed on Ru^δ+^ species react with O_2_^−^ species to produce CO_2_ and H_2_O, which is a Langmuir-Hinshelwood mechanism.

## Conclusions

A series of Ru/Sn_*x*_Ti_1−*x*_O_2_ catalysts were prepared by a one-step hydrothermal method for the catalytic oxidation of CO and C_3_H_8_. The preparation conditions of Ru/Sn_*x*_Ti_1−*x*_O_2_ catalysts were optimized for CO oxidation reaction. Ru/Sn_0.67_Ti_0.33_O_2_ catalyst shows best CO catalytic activity and stability at low temperature under the condition of hydrothermal temperature at 180 °C, hydrothermal time at 24 h, and calcination temperature at 400 °C.

The effects of different molar ratios of Sn/Ti on the catalytic properties of Ru/Sn_*x*_Ti_1−*x*_O_2_ catalysts for CO and C_3_H_8_ were investigated under the optimum preparation conditions. The results show that the Ru/Sn_0.67_Ti_0.33_O_2_ catalyst exhibits better low-temperature activity and stability. The conversion of CO reached 90% at 240 °C, and *T*_50_ of which keeps at 180 °C. The complete conversion of C_3_H_8_ could be achieved at 500 °C, and its *T*_50_ remains at 320 °C. The excellent catalytic activity of Ru/Sn_0.67_Ti_0.33_O_2_ catalyst is attributed to the factors listed as follows.

1. The successful incorporation of Sn^4+^ into the TiO_2_ lattice to replace Ti^4+^ forms a homogeneous solid solution (–Sn^4+^–O–Ti^4+^– species), which enhances the interaction between active component Ru and carrier Sn_*x*_Ti_1−*x*_O_2_. The crystal growth of the anatase phase can be inhibited by the introduction of Sn^4+^, which results in the presence of the rutile phase.
2. Ultrafine Ru nanoparticles (~ 5 nm) are highly dispersed on Sn_*x*_Ti_1−*x*_O_2_ support, suggesting that the introduction of Sn^4+^ could not only prevent grain agglomeration and induce a smaller grain size, but also produce more defects such as oxygen vacancies.
3. CO and C_3_H_8_ species can be absorbed on Ru^δ+^ sites; O_2_^−^ is formed by the adsorption of O_2_ on the oxygen vacancies. The adsorbed CO and C_3_H_8_ react with O_2_^−^ to produce CO_2_ and H_2_O.

## Supplementary information

**Additional file 1: Figure S1** Effect of different hydrothermal temperature on Ru/Sn_0.67_Ti_0.33_O_2_ catalytic CO. **Figure S2** Effect of different hydrothermal time on Ru/Sn_0.67_Ti_0.33_O_2_ catalytic CO. **Figure S3** Effect of different calcination temperature on Ru/Sn_0.67_Ti_0.33_O_2_ catalytic CO. **Table S1** Catalytic activity comparison of different catalysts for CO oxidation. **Table S2** Catalytic activity comparison of different catalysts for C_3_H_8_ oxidation

## Data Availability

All data generated or analyzed during this study are included in this published article and supporting information.

## Abbreviations

XRD: X-ray diffraction
BET: Brunauer-Emmett-Teller
FT-IR: Fourier transform infrared
TEM: Transmission electron microscopy
XPS: X-ray photoelectron spectroscopy
H_2_-TPR: H_2_-temperature-programmed reduction
O_2_-TPD: Temperature-programmed oxygen desorption
DOC: Diesel oxidation catalysts
SCR: Selective catalytic reduction
DPF: Diesel particulate filter
SOF: Soluble organic fraction
