# Supplementary material for: A Novel One-Step Hydrothermal Preparation of Ru/SnxTi1−xO2 Diesel Oxidation Catalysts and its Low-Temperature Performance
Source: Nanoscale Res Lett. 2020 May 14;15:109. doi: 10.1186/s11671-020-03339-4 (PMC7225244; doi:10.1186/s11671-020-03339-4)
Supplement: Supplementary file 1 — Additional file 1: Figure S1 Effect of different hydrothermal temperature on Ru/Sn0.67Ti0.33O2 catalytic CO. Figure S2 Effect of different hydrothermal time on Ru/Sn0.67Ti0.33O2 catalytic CO. Figure S3 Effect of different calcination temperature on Ru/Sn0.67Ti0.33O2 catalytic CO. Table S1 Catalytic activity comparison of different catalysts for CO oxidation. Table S2 Catalytic activity comparison of different catalysts for C3H8 oxidation [file 11671_2020_3339_MOESM1_ESM.pdf]

Supporting Information

A novel one-step hydrothermal preparation of Ru/$Sn_xTi_{1-x}O_2$ Diesel Oxidation Catalysts and Its Low Temperature Performance

Li Fan, Qi Sun , Wei Zheng , Qinyuan Tang , Ting Zhang , Mengkui Tian *

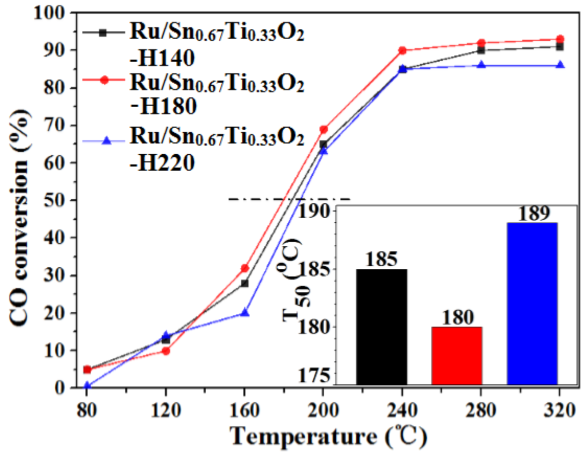

Fig. S1 Effect of different hydrothermal temperature on Ru/$Sn_{0.67}Ti_{0.33}O_2$ catalytic CO

Fig.S1 shows the catalytic activities of CO oxidation on the Ru/$Sn_{0.67}Ti_{0.33}O_2$ catalysts under the different hydrothermal temperature (140 °C, 180 °C and 220 °C) while the hydrothermal time is 24 h and calcination temperature is 400 °C. It can be seen that catalytic performances of Ru/$Sn_{0.67}Ti_{0.33}O_2$ catalysts increased firstly and then tended to be stabilized with the increase of reaction temperature. The T_{50} of Ru/$Sn_{0.67}Ti_{0.33}O_2$ catalyst to oxidize CO is 180 °C when the hydrothermal temperature at 180 °C, which is lower reaction temperature than other hydrothermal temperature and the conversion of CO reaches 90% at 240 °C. It is attributed to the increase of hydrothermal temperature is beneficial to the growth of solid solution grains, but the excessive hydrothermal temperature will lead to overgrowth of grains and ruduce the catalytic oxidation performance. Therefore, the optimal hydrothermal temperature is determined to be 180 °C.

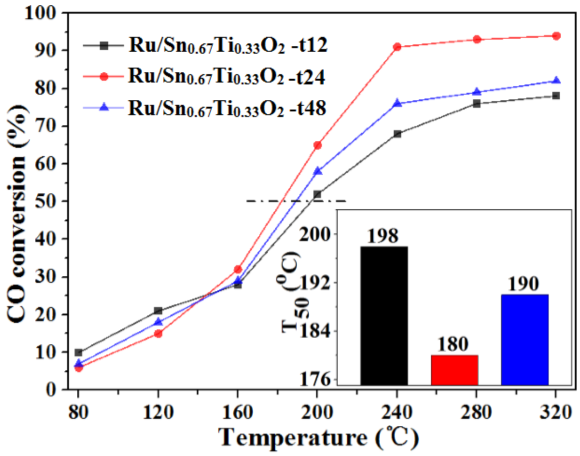

Fig. S2 Effect of different hydrothermal time on $Ru/Sn_{0.67}Ti_{0.33}O_2$ catalytic CO

Fig.S2 shows the catalytic activities of CO oxidation on the $Ru/Sn_{0.67}Ti_{0.33}O_2$ catalyst under the different hydrothermal time (12 h, 24 h and 48 h) while the hydrothermal temperature is 180 ^{o}C and calcination temperature is 400 ^{o}C. It shows that catalytic performances of $Ru/Sn_{0.67}Ti_{0.33}O_2$ catalysts increased first and then tended to be stabilized with the increase of reaction temperature. The T_{50} of $Ru/Sn_{0.67}Ti_{0.33}O_2$ to CO is 180 ^{o}C when the hydrothermal time at 24 h, which is lower reaction temperature than other hydrothermal time and the conversion of CO reached 90% at 240 ^{o}C. This phenomenon suggests that the increase of hydrothermal time could promote the growth of grains, but too long hydrothermal time will lead to the aging of grains and the ruduction of catalytic oxidation efficiency.

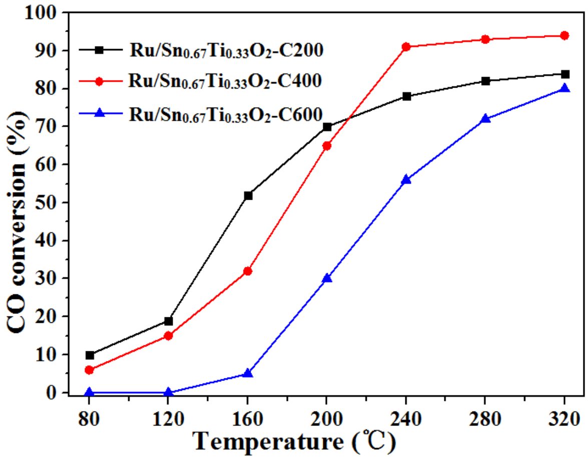

Fig. S3 Effect of different calcination temperature on $Ru/Sn_{0.67}Ti_{0.33}O_2$ catalytic CO

Fig.S3 shows the catalytic activities of CO oxidation on the $Ru/Sn_{0.67}Ti_{0.33}O_2$ catalyst under the different calcination temperature (200 ^{o}C, 400 ^{o}C and 600 ^{o}C) while the hydrothermal temperature is 180 ^{o}C and hydrothermal time is 24 h. It can be seen that catalytic performances of $Ru/Sn_{0.67}Ti_{0.33}O_2$ catalysts increased first and then tended to be flat with the increase of reaction temperature. Before reaction

temperature at 200 ^oC, the activity of $Ru/Sn_{0.67}Ti_{0.33}O_2$ decrease in the order of $Ru/Sn_{0.67}Ti_{0.33}O_2$-C200 > $Ru/Sn_{0.67}Ti_{0.33}O_2$-C400 > $Ru/Sn_{0.67}Ti_{0.33}O_2$-C600. While after reaction temperature 200 ^oC, the activity of $Ru/Sn_{0.67}Ti_{0.33}O_2$ decrease in the order of $Ru/Sn_{0.67}Ti_{0.33}O_2$-C400 > $Ru/Sn_{0.67}Ti_{0.33}O_2$-C200 > $Ru/Sn_{0.67}Ti_{0.33}O_2$-C600 as shown in Fig.S3. This is because the actual value of O/Ru is higher than the theoretical value at 250-700 ^oC, Ru is easier to oxidize to RuO_x. When the calcination temperature is 400 ^oC, the conversion of CO reached 90% at 240 ^oC. Therefore, the optimal calcination temperature is determined to be 400 ^oC.

According to the above analysis, the preparation conditions of $Ru/Sn_{0.67}Ti_{0.33}O_2$ catalysts including hydrothermal temperature, hydrothermal time and calcination temperature were optimized with CO oxidation. The catalytic activity of $Ru/Sn_{0.67}Ti_{0.33}O_2$ for CO is excellent under the conditions of the hydrothermal temperature at 180 ^oC, the hydrothermal time at 24 h and calcination temperature at 400 ^oC, the T_{50} of $Ru/Sn_{0.67}Ti_{0.33}O_2$ is 180 ^oC. When the reaction temperature is 240 ^oC, the conversion of $Ru/Sn_{0.67}Ti_{0.33}O_2$ to CO reaches 90%.

Table S1 Catalytic activity comparison of different catalysts for CO oxidation

Catalyst	Preparation method	T_{50} (^oC)	Space velocity (h^{-1})	Ref
Ru/C12Al7:O^{2-}	solid-state reaction	180	25200	[6]
Pt/Al_2O_3	impregnation	230	17000	[30]
Pt/ZnO	impregnation	245	30000	[30]
Pt/TiO_2	Co-precipitation impregnation	204	60000	[4]
$8\%CuO/SnO_2$	Sol-gel dispersion-precipitation	270	20040	[15]
TiO_2-SnO_2	Sol-gel dispersion-precipitation	240	20000	[31]
5CuT1S3	Co-precipitation impregnation	185	30000	[5]
$Ce_{0.9}Zr_{0.1}O_2$	hydrothermal	250	43200	[22]
$Ce_{0.8}Zr_{0.2}O_2$	hydrothermal	300	43200	[22]
$Ce_{0.7}Zr_{0.3}O_2$	hydrothermal	280	43200	[22]
Ce-Ti	Co-precipitation	348	30000	[27]
$Ru/Sn_{0.67}Ti_{0.33}O_2$	hydrothermal impregnation	180	60000	This work

Table S2 Catalytic activity comparison of different catalysts for C_3H_8 oxidation

Catalyst	Preparation method	T_{50} (^oC)	Space velocity(h^{-1})	Ref
$0.5\%Ru/ZnAl_2O_4$	co-precipitation	480	32000	[28]
1% $Ru/ZnAl_2O_4$	co-precipitation	500	32000	[28]
1.5% $Ru/ZnAl_2O_4$	co-precipitation	540	32000	[28]
0.3Pd-0.7Pt/γ-Al_2O_3	impregnation	360	50000	[29]

Catalyst	Preparation method	T_{50} (ºC)	Space velocity(h^{-1})	Ref
0.7Pd-0.3Pt/γ-Al_2O_3	impregnation	325	50000	[29]
Pt/CeO_2	co-precipitation	620	50000	[32]
Pt/CeO_2	hydrothermal	675	50000	[32]
Pt/Co_3O_4	co-precipitation	447	6000	[33]
Pd/Al_2O_3	impregnation	411	30000	[34]
PtPd/Al_2O_3	impregnation	428	30000	[34]
PtPd/$CeAl_2O_3$	impregnation	436	30000	[34]
Ru/$Sn_{0.67}Ti_{0.33}O_2$	hydrothermal	320	60000	This work
